# Not All That Itches Is DRESS: A Case Report of Lichenified Dermatosis and Hypereosinophilia Associated With Tocilizumab

**DOI:** 10.1155/crii/8016268

**Published:** 2026-07-17

**Authors:** Mani Maheshwari, Brian Swick, Benjamin Davis, Petar Lenert

**Affiliations:** ^1^ Division of Immunology, Department of Internal Medicine, Carver College of Medicine, University of Iowa Hospitals and Clinics, Iowa City, Iowa, USA, uihealthcare.org; ^2^ Department of Dermatology, Carver College of Medicine, University of Iowa Hospitals and Clinics, Iowa City, Iowa, USA, uihealthcare.org

**Keywords:** anti-IL-6 receptor antibody, case report, DRESS, eosinophilia

## Abstract

Drug‐induced hypereosinophilic syndromes (HESs) have been associated with a variety of medications, including biologic agents. We present a case of an 85‐year‐old female with a history of giant cell arteritis (GCA) complicated by ascending aortic aneurysm and aortitis, who developed marked peripheral eosinophilia (peaking at 3710/µL) and a persistent pruritic papular rash during long‐term tocilizumab therapy. Extensive infectious, hematologic, and immunologic evaluations were unrevealing. While the clinical picture raised concern for a drug‐induced hypersensitivity reaction, histopathology was more consistent with lichen simplex chronicus, and there was no evidence of systemic organ involvement. Her eosinophilia and cutaneous symptoms improved significantly after discontinuing tocilizumab, implicating the drug as a possible trigger. This case highlights the diagnostic challenges in distinguishing drug reaction with eosinophilia and systemic symptoms (DRESS) from other drug‐induced or idiopathic dermatoses in patients receiving biologic therapy and underscores the importance of clinicopathologic correlation in guiding management.

## 1. Introduction

Hypereosinophilic syndromes (HESs) encompass a spectrum of conditions ranging from mild peripheral blood eosinophilia to life‐threatening end‐organ damage. Drug‐induced eosinophilia may manifest as distinct clinical entities depending on the offending agent. Leukotriene receptor antagonists, such as montelukast, have been implicated in eosinophilic granulomatosis with polyangiitis (EGPA), a systemic necrotizing vasculitis [1]. By contrast, drug reaction with eosinophilia and systemic symptoms (DRESS) is most frequently associated with a different class of culprits, including allopurinol, aromatic antiepileptics (carbamazepine, phenytoin, and lamotrigine), antituberculous agents, and specific antibiotics such as vancomycin and sulfonamides [2].

Tocilizumab, an IL‐6 receptor antagonist, is widely used for the treatment of autoimmune diseases such as rheumatoid arthritis and giant cell arteritis (GCA) [3, 4]. The most commonly observed adverse effects occurring during treatment with anti‐IL‐6 receptor antagonists include neutropenia, lipid abnormalities, and elevated liver enzymes [3, 4]. While tocilizumab‐induced DRESS‐like syndrome is a rare complication, several cases have been reported in the literature, with outcomes ranging from reversible cutaneous manifestations to fatal outcomes due to multiorgan failure [5, 6]. DRESS is a severe, potentially life‐threatening type IVb T cell–mediated hypersensitivity reaction characterized by fever, rash, eosinophilia, and internal organ involvement, with a mortality rate of 1.7%–10% as described in the prospective RegiSCAR study by Kardaun et al. [2].

The present case is notable for several reasons. First, it illustrates the diagnostic difficulty of distinguishing true DRESS from a tocilizumab‐associated dermatosis in a clinical context where both are plausible, a distinction with meaningful therapeutic implications. Second, the prolonged latency of approximately 2 years between initiation of tocilizumab and symptom onset is atypical for classic DRESS, which usually manifests within 2 to 8 weeks of drug exposure. Third, the final histopathologic diagnosis of lichen simplex chronicus, a finding attributed to chronic pruritus rather than a primary drug hypersensitivity, underscores the importance of tissue confirmation and formal diagnostic scoring in cases where clinical suspicion alone may be misleading. Finally, this case highlights the need for heightened dermatologic vigilance in elderly patients on long‐term biologic immunosuppression. Herein, we present a case of reversible HES in an elderly patient with GCA associated with treatment with the anti‐IL‐6 receptor antagonist tocilizumab.

## 2. Case Presentation

An 82‐year‐old woman was referred for rheumatologic evaluation following ascending aortic aneurysm repair, after surgical pathology revealed findings consistent with old‐healed aortitis and focal residual inflammation. She had undergone an ascending aortic aneurysm replacement with a graft for a 5.4 cm ascending aortic aneurysm with mild aortic insufficiency and risk for dissection. Her postoperative course was notable for a pleural effusion requiring thoracentesis and transient diastolic dysfunction requiring diuresis. Histopathology demonstrated evidence of both old‐healed aortitis with intimal fibrosis, medial scarring, and focal residual lymphoplasmacytic infiltrates and active inflammatory infiltrates that were sparse and patchy. Prior to surgery, she was in her usual state of health, reporting only mild dyspnea on exertion and transient temporomandibular joint and posterior auricular pain that had self‐resolved years earlier. She did not report fevers, chills, night sweats, scalp tenderness, jaw claudication, hoarseness of voice, limb claudication, unexplained weight loss, or polymyalgia‐type symptoms.

**Figure 1:**
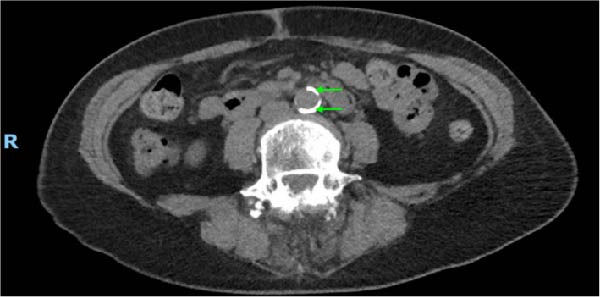
PET/CT F‐18 FDG scan showing mild circumferential low‐grade uptake surrounding the descending aorta.

Given the histopathologic evidence of aortitis and clinical suspicion for large‐vessel vasculitis, a PET/CT F‐18 FDG scan from skull base to mid‐thigh was performed to evaluate for vascular FDG uptake consistent with GCA. The scan revealed mild circumferential low‐grade uptake surrounding the descending aorta and bilateral subclavian arteries (Figure 1), a distribution characteristic of large‐vessel GCA involvement [4]. These findings, in combination with the histologic evidence of aortitis, the patient’s advanced age, and the previously noted constitutional symptoms, were consistent with subclinical large‐vessel GCA. Further laboratory assessment revealed persistently elevated inflammatory markers (erythrocyte sedimentation rate [ESR] of 116 [*N* = 0–20 mm/h] and C‐reactive protein [CRP] of 4.9 [*N* < 0.5 mg/dL]), suggesting ongoing systemic inflammation. Soon after this she was started on a prednisone burst and taper followed by monthly tocilizumab IV infusions. Her ESR and CRP levels normalized, and she entered clinical remission.

After being on tocilizumab infusions for approximately 2 years, she presented to the rheumatology clinic with new‐onset skin rash and elevated eosinophil blood counts. Initial eosinophil count was 1040/µL (*N* = 40–390/µL), peaking at 3710/µL in December 2024. The rash began as an itchy, burning papular eruption on her arms, later spreading to her trunk and legs.

A systematic evaluation for alternative etiologies of eosinophilia was pursued. Testing for ANA and ANCA was performed to assess for an autoimmune or vasculitic process; parasitic serologies were obtained to exclude helminthic infection, a classic cause of peripheral eosinophilia; fungal cultures were sent to rule out disseminated fungal infection. Serum tryptase was measured to evaluate for mastocytosis, and vitamin B12 levels were checked given their elevation in myeloproliferative conditions and primary HES. Flow cytometry was performed to assess for an underlying hematologic malignancy or clonal T‐cell disorder driving the eosinophilia. All of these investigations were unrevealing. A small subset of CD56+ monocytes was noted on flow cytometry, of uncertain clinical significance.

Given the temporal relationship between tocilizumab administration and symptom onset and the absence of alternative causes, DRESS‐like syndrome was suspected. To formally assess the likelihood of DRESS, the RegiSCAR scoring system was applied [2]. The patient did not have documented fever ≥ 38.5°C (0 points), lymphadenopathy (0 points), or atypical lymphocytes on peripheral smear (0 points). Eosinophilia was present and peaked above 1500/µL, qualifying for the highest eosinophilia score (+2 points). The rash was a pruritic papular eruption involving the arms, trunk, and legs, though it did not cover more than 50% of body surface area, lacked the edematous or infiltrated morphology typical of DRESS, and was not associated with facial edema. There was no evidence of internal organ involvement, including normal liver enzymes and renal function (0 points). Resolution occurred over more than 15 days following drug withdrawal. The aggregate RegiSCAR score was consistent with a “possible” DRESS reaction at best, falling short of the “probable” or “definite” thresholds. This formal scoring, combined with the histopathologic finding of lichen simplex chronicus rather than a drug hypersensitivity pattern, supported the conclusion that this presentation did not fulfill criteria for true DRESS.

**Figure 2:**
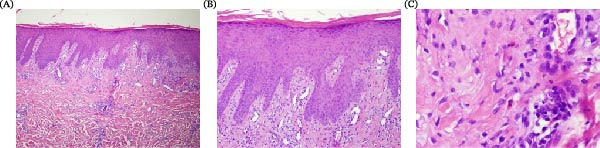
Histopathology demonstrated psoriasiform epidermal hyperplasia with minimal spongiosis and scant superficial perivascular lymphocytic infiltrate with rare eosinophils consistent with lichen simplex chronicus. (A) 40×; (B) 100×; and (C) 400×.

**Figure 3:**
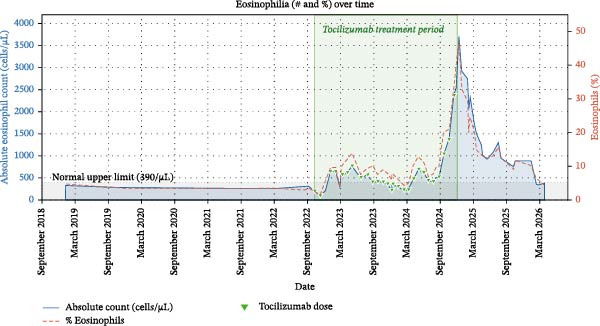
Eosinophil count trends over time (2019–2026): serial measurements of absolute and relative eosinophil counts demonstrating a peak during tocilizumab therapy and subsequent decline postdiscontinuation. Note that in December 2024, eosinophils reached the highest point (3710/µL), followed by a gradual decline and normalization.

Tocilizumab was discontinued in December 2024. A skin biopsy was pursued, which revealed histopathology more consistent with lichen simplex chronicus (Figure 2). Symptomatic treatment included oral antihistamines and topical corticosteroids (triamcinolone, followed by clobetasol). No systemic steroids were initiated due to the overall stable systemic status. After drug withdrawal, eosinophil counts started to decline from 3710/µL in December 2024 to 2340/µL by February 2025, with continued improvement over subsequent months (Figure 3). The most recent eosinophil count was 750/µL. Her GCA remained in stable remission without a need for further immunosuppressive therapy. The rash gradually resolved without recurrence over the following 9 months.

## 3. Discussion

DRESS should be considered in patients on tocilizumab treatment who develop new‐onset skin rash and eosinophilia [4–6]. Reported literature cases demonstrate a variety of clinical manifestations, including fever, lymphadenopathy, hepatitis, gastrointestinal symptoms, and, rarely, life‐threatening multiorgan involvement [4–6]. The pathophysiology of drug‐induced DRESS‐like syndrome likely results from altered cytokine balance due to IL‐6 blockade promoting TH2‐mediated eosinophilic pathways [2]. True DRESS syndrome with tocilizumab remains rare, and most reported cases feature more severe systemic involvement and classic rash morphology.

Beyond IL‐6 inhibitors, a wide spectrum of other medications, including antibiotics, antiepileptics, and other biologics (canakinumab, rilonacept, daclizumab, and anakinra), have been implicated in multisystem hypersensitivity reactions or drug‐induced HESs [7]. In the systematic review of 196 cases, Bartal et al. [8] observed that almost every major pharmacologic class of medications could trigger eosinophil‐mediated organ injury, highlighting the importance of careful review of medications in any patient with new‐onset eosinophilia with or without associated systemic manifestations.

Emerging data suggest that in patients with large‐vessel GCA, including those with aortitis, combination therapy with tocilizumab and methotrexate may improve disease control and permit shorter durations or lower cumulative exposure to tocilizumab, potentially reducing treatment‐related adverse events. Recent studies have reported that MTX cotherapy may allow safe tapering or discontinuation of tocilizumab in selected patients while maintaining remission, an approach that may be particularly attractive in elderly populations vulnerable to biologic toxicity [9, 10]. In the present case, the patient received tocilizumab for approximately 2 years—a duration exceeding the 12–18 months typically recommended before considering tapering in patients achieving clinical remission [4]. Throughout this period, she maintained clinical and laboratory remission, with normalized ESR and CRP, without evidence of active GCA relapse. The extended treatment course was driven by clinician preference for stability in a high‐risk patient given her history of large‐vessel aortitis and aortic aneurysm repair. In retrospect, the durable remission observed following tocilizumab cessation—necessitated here by toxicity rather than planned tapering—supports the concept that prolonged biologic therapy may not be required indefinitely in all patients who achieve sustained remission. In addition, although cutaneous manifestations are uncommon in GCA, a broad spectrum of skin findings, including pruritic eruptions and secondary lichenification, has been described, underscoring the importance of considering both disease‐related and treatment‐related etiologies when evaluating new dermatologic findings in this population [11]. Beyond GCA‐specific skin manifestations, chronic immunosuppression itself carries a recognized burden of cutaneous adverse effects, and prolonged pruritus induced by immunologic dysregulation can lead to lichenification through the itch‐scratch cycle, independent of any primary drug hypersensitivity [7, 12].

## 4. Conclusion

Clinicians should be aware that tocilizumab and other biologics can be associated with peripheral eosinophilia and pruritic dermatoses, which may mimic but do not always fulfill criteria for DRESS. Careful clinicopathologic correlation is essential, and drug discontinuation may be warranted in cases of persistent or unexplained eosinophilia and rash, even in the absence of classic DRESS features.

## Funding

No funding was received for this manuscript.

## Consent

No written consent has been obtained from the patients as there is no patient‐identifiable data included in this case report.

## Conflicts of Interest

The authors declare no conflicts of interest.

## Data Availability

The data that support the findings of this study are available upon request from the corresponding author. The data are not publicly available due to privacy or ethical restrictions.
